# Circulating Soluble CD36 is Similar in Type 1 and Type 2 Diabetes Mellitus versus Non-Diabetic Subjects

**DOI:** 10.3390/jcm8050710

**Published:** 2019-05-18

**Authors:** Esmeralda Castelblanco, Lucía Sanjurjo, Mireia Falguera, Marta Hernández, José-Manuel Fernandez-Real, Maria-Rosa Sarrias, Nuria Alonso, Didac Mauricio

**Affiliations:** 1 Department of Endocrinology & Nutrition, University, Hospital de la Santa Creu i Sant Pau & Institut d’Investigació Biomèdica Sant Pau (IIB Sant Pau), 08041 Barcelona, Spain; esmeraldacas@gmail.com; 2 Centre for Biomedical Research on Diabetes and Associated Metabolic Diseases (CIBERDEM), 08907 Barcelona, Spain; 3 Innate Immunity Group, Health Sciences Research Institute Germans Trias i Pujol, 08916 Badalona, Spain; lsanjurjo@igtp.cat (L.S.); mrsarrias@igtp.cat (M.-R.S.); 4 Primary Health Care Cervera, Gerència d’Atenció Primaria, Institut Català de la Salut, Unitat de Suport a la Recerca, Institut Universitari d’Investigació en Atenció Primària Jordi Gol (IDIAP Jordi Gol), 25200 Cervera, Spain; mireiafalguera@hotmail.com; 5 Biomedical Research Institute of Lleida (IRBLleida) & University of Lleida, 25198 Lleida, Spain; 6 Department of Endocrinology & Nutrition, University Hospital Arnau de Vilanova & Biomedical Research Institute of Lleida (IRBLleida), 25198 Lleida, Spain; martahernandezg@gmail.com; 7 Department of Diabetes, Endocrinology & Nutrition, Hospital Dr Josep Trueta & Biomedical Research Institute of Girona (IDIBGI), 17007 Girona, Spain; jmfreal@idibgi.org; 8 Centre for Biomedical Research on Physiopathology of Obesity and Nutrition (CIBEROBN), 17007 Girona, Spain; 9 Centre for Biomedical Research on Liver and Digestive Diseases (CIBEREHD), 28029 Madrid, Spain; 10 Department of Endocrinology & Nutrition, University Hospital Germans Trias i Pujol & Health Sciences Research Institute, 08916 Badalona, Spain

**Keywords:** sCD36, type 1 diabetes mellitus, type 2 diabetes mellitus

## Abstract

The aim of this study was to determine whether plasma concentrations of sCD36 (soluble CD36) are associated with the presence of type 1 or type 2 diabetes. Plasma levels of sCD36 were analysed in 1023 subjects (225 type 1 diabetes (T1D) patients, 276 type 2 diabetes (T2D) patients, and 522 non-diabetic control subjects) using an enzyme-linked immunosorbent assay (ELISA). Multinomial and logistic regression models were performed to evaluate associations with sCD36 and its association with diabetes types. There were no significant differences in sCD36 (*p* = 0.144) among study groups, neither in head-to-head comparisons: non-diabetic versus T1D subjects (*p* = 0.180), non-diabetic versus T2D subjects (*p* = 0.583), and T1D versus T2D patients (*p* = 0.151). In the multinomial model, lower sCD36 concentrations were associated with older age (*p* < 0.001), tobacco exposure (*p* = 0.006), T2D (*p* = 0.020), and a higher-platelets count (*p* = 0.004). However, in logistic regression models of diabetes, sCD36 showed only a weak association with T2D. The current findings show a weak association of circulating sCD36 with type 2 diabetes and no association with T1D.

## 1. Introduction

CD36 belongs to the class B scavenger receptor family, it is a transmembrane glycoprotein of 88-kDa expressed in a wide variety of cell types [1]. CD36 recognises many types of ligands, such as oxidised low-density lipoprotein (oxLDL), advanced glycation products, free fatty acids, or apoptotic cell surfaces [2,3,4,5]. This multivariate ligand recognition allows it to exert several functions, depending on the cell type. Among others, CD36 internalise modified lipoproteins (e.g., oxidised LDL), which facilitates cholesterol accumulation in macrophages or the regulation of fatty acid uptake through the plasma membrane and the subsequent metabolism [6,7]. CD36 expression or function are involved in angiogenesis, macrophage and platelet activation, lipid metabolism, and inflammation [8,9,10,11], which influences susceptibility to certain metabolic diseases, such as obesity, insulin resistance, impaired glucose tolerance, and fatty liver disease [7,12,13]. CD36 has been reported to be associated with type 2 diabetes (T2D) [14]. In monocytes and macrophages, CD36 is up-regulated by hyperglycaemia, insulin resistance, and oxLDL [8,10,15,16,17]. Moreover, oxLDL concentrations are elevated in type 2 diabetes [18,19].

The soluble form of CD36 (sCD36) was first described to be associated with metabolic syndrome in a small group of subjects: sCD36 was elevated in T2D patients compared with both lean (5-fold) and obese (2- to 3-fold) non-diabetic subjects [20]. Moreover, it has been described to be associated with insulin resistance, impaired glucose tolerance, and fatty liver in non-diabetic subjects [21,22,23]. Other studies have found discordant results concerning the circulating concentrations of sCD36 in patients with T2D, resulting in no difference [24,25], or increased concentrations in T2D diabetic subjects with chronic kidney disease [26], compared to non-diabetic subjects. However, a recent study found no association of sCD36 with glucose or additional factors that were described in previous studies to be associated with its concentrations [24]. To our knowledge, no studies have assessed sCD36 concentrations in patients with type 1 diabetes (T1D). 

The aim of the current study was to assess whether the circulating concentrations of sCD36 are associated with diabetes, T1D or T2D, compared with a non-diabetic control group, all of them without previous cardiovascular events and free from chronic kidney disease. Taken together, the findings show a weak association of circulating sCD36 with T2D and no association with T1D. 

## 2. Experimental Section

### 2.1. Subjects 

One thousand and twenty-three patients were recruited from the University Hospitals Arnau de Vilanova (Lleida, Spain) and Germans Trias i Pujol (Badalona, Spain) to participate in this study. Subjects with T1D (*n* = 225) and T2D (*n* = 276) were selected from two previous studies [27,28]. The control group (*n* = 522) was selected from a population-based study in our region (subjects without diabetes on the basis of values of HbAc1 <6.5% and glucose <126 mg/dL) [29]. Additionally, 50 subjects, 22 T2D patients and 28 non-diabetic subjects, were recruited at the University Hospital Germans Trias i Pujol to perform the sub-study using flow cytometric analysis. The inclusion criteria for the four groups were as follows: age range 20–85 years, absence of established chronic kidney disease (defined as calculated glomerular filtration rate <60 mL/min and/or urine albumin/creatinine ratio >299 mg/g), and absence of known clinical cardiovascular events or associated revascularization procedures, including coronary heart disease, cerebrovascular disease, or peripheral vascular disease (including the diagnosis of diabetic foot disease). 

Blood samples were collected in the fasting state, and blood tests were performed using standard laboratory methods [28]. Urine tests were performed in diabetic patients following standard laboratory methods. Subjects were considered to have hypertension or dyslipidaemia if they were under anti-hypertensive or lipid-lowering agent treatment, respectively. Blood samples for sCD36 measurements were collected in the fasting state with EDTA tubes, processed immediately after extraction, and stored at −80 °C at the biobanks of the participant centres until determination. Peripheral blood samples for flow cytometric and real-time PCR assays were collected on the same condition and processed after one hour on a shaker plate. Local ethics committees of both participating centres approved the study (12/2009 and P11/11) which followed the Declaration of Helsinki. All participants provided written informed consent before inclusion. 

### 2.2. Determination for sCD36 by ELISA

Plasma concentrations of human sCD36 were measured using a commercially available ELISA kit (Nordic BioSite, Täby, Sweden), in accordance with the manufacturer’s instructions. Appropriate dilutions of patient samples were measured in duplicates. Briefly, standards and samples were pipetted into the wells and incubated for 2 h on the plate shaker at room temperature (RT). After washing 4 times, a detection antibody was added and incubated for 2 h on the plate shaker at RT. Following washing 4 times, a streptavidin conjugated with Horseradish Peroxidase solution was added to the wells and incubated for 50 min on the plate shaker at RT and protected from light. After washing 4 times, 50 μL substrate solution (TMB-tetramethylbenzidine) was added to the wells and incubated for 20 min at RT protected from light. The colour development was stopped with 50 μL of stop solution. Absorbance was read at 450 nm using SpectraMax 340PC384 (Molecular Devices, LLC, Sunnyvale, California, USA). The results were analysed using a log–log curve fit. The calibration was performed with recombinant human CD36 in the concentration range 1.95–250 ng/mL. For sCD36 concentrations lower than the detection limit, a value of 0.05 ng/mL was assigned (in the non-diabetic control group, *n* = 64/522; T1D, *n* = 41/225; T2D, *n* = 24/276). Intra-assay and inter-assay precision coefficients of the ELISA assay were 4%–6% and 8%–12% respectively, as determined by the manufacturer. 

### 2.3. Consistency Test of sCD36 Determination

Due to the variability found in the concentrations of sCD36 in the published articles, we wanted to rule out that this was a consequence of differences in pre-analytical conditions. We designed an experiment to compare sCD36 assessed by ELISA (Nordic BioSite, Täby, Sweden) that included 96 samples from 14 volunteers. Plasma samples were processed at different centrifugation velocities: 1500× *g* (following our protocol), 1850× g and 3000× g (velocities used in other studies) [20,30]. Moreover, it was assessed whether the number of freeze-thaw cycles affected the sCD36 concentration. Samples without previous thaw, as used in our protocol, and with three freeze-thaw cycles, as requested by the in-house ELISA found in the literature, were evaluated [31].

### 2.4. Flow Cytometric Analysis 

The analysis of CD36 by flow cytometry included 50 subjects, 22 T2D patients and 28 non-diabetic subjects. Peripheral blood samples were collected in the fasting state into vacutainers (BD Bioscience, San Jose, CA, USA) containing EDTA. Erythrocytes were lysed with ammonium chloride (BD Pharm Lyse™, San Jose, CA, USA), then the cells were washed with phosphate buffered saline (PBS), and incubated with monoclonal antibodies against CD36 (Miltenyi Biotec, Bergisch Gladbach, Germany), CD3, and CD14 (BD Biosciences, San Jose, CA, USA). Thereafter, flow cytometric analysis was carried out on a Fortessa SORP flow cytometer (BD Biosciences, San Jose, CA, USA), using sample acquisition and analysis software FACSDiva v6.2 (BD Biosciences, San Jose, CA, USA). 

### 2.5. Real-Time PCR 

The analysis of CD36 mRNA included the above-mentioned 50 subjects (22 T2D patients and 28 without diabetes). Erythrocytes were lysed with ammonium chloride (BD Pharm Lyse™), then the cells were washed with PBS and disrupted with QUIzol Lysis Reagent (Quiagen, Hilden, Germany), and total RNA was extracted using the miRNA Mini Kit (Quiagen, Hilden, Germany). Total RNA (1 µg) was reverse-transcribed using the Transcriptor First Strand cDNA Synthesis Kit (Roche, Basel, Switzerland). Each reaction was then amplified in a LightCycler^®^ 480 PCR system using SYBR Green I Master (Roche, Basel, Switzerland). The primer pairs used were: forward primer 5′->3′ (GAGAACTGTTATGGGGCTAT) and reverse primer 5′->3′ (TTCAACTGGAGAGGCAAAGG). Gene expression values were normalized to the expression levels of glyceraldehyde 3-phosphate dehydrogenase (GAPDH). 

### 2.6. Statistical Analyses

Descriptive statistics of mean (standard deviation) or median (interquartile range) were estimated for quantitative variables while, for qualitative variables, absolute and relative frequencies were used. The differences between groups T1D, T2D, and control group, were assessed by Student’s *t*-test, ANOVA, the Mann–Whitney test, or the Kruskal–Wallis test. Regarding the differences in qualitative variables, this was assessed by the Chi-squared test or Fisher’s exact test. Tukey’s correction was used to account for multiple tests. Correlations were assessed by Spearman’s rank correlation coefficient. Associations between qualitative variables and sCD36 concentration were tested with the *T* test. Multinomial logistic regression models of sCD36 quartile were performed to study the variables associated with their levels, first quartile was used as reference. In these models, variables of the bivariate analysis with a *p* value <0.2 and clinical relevance were used. Logistic regression models of T1D versus non-diabetic and T2D versus non-diabetic were performed. For the models of diabetes, a non-diabetic age- and sex-matched group from the non-diabetic participants (*n* = 522) were selected for T1D (*n* = 314) and T2D (*n* = 247). In all models, the goodness-of-fit assumption was tested by the Hosmer–Lemeshow test. Logistic regression models were checked by receiver-operating characteristic (ROC) curves and DeLong’s test to correlated ROC curves. Estimates of odds ratios was reported with corresponding 95% confidence intervals and statistical significance was established as a *p*-value <0.05. The R statistical software, version 3.3.1, and SPSS software (version 22, IBM, SPSS, Chicago, IL, USA) were used for all of the analyses.

## 3. Results

A total of 1023 individuals, clinically well-characterised (225 type 1 diabetic patients, 276 type 2 patients, and 522 non-diabetic subjects), were included in the study. In the overall study population, the mean age was 50 years, and up to 45.4% were men. The age in the different groups of participants ranged between 23 and 77 years in patients with T1D, between 40 and 75 years in patients with T2D, and between 27 and 84 years in the non-diabetic control group. There were significant differences among the three study groups for all studied variables, except for tobacco exposure. All the clinical characteristics can be found in Table 1.

### 3.1. Circulating sCD36 in the Study Groups

There were no significant differences in the median plasma concentrations of sCD36 among the three study groups (*p* = 0.144). In head-to-head comparisons, concentrations of sCD36 were not different between non-diabetic and T1D subjects (2.84 ng/mL versus 3.66 ng/mL; *p* = 0.180), non-diabetic and T2D subjects (2.84 ng/mL versus 2.62 ng/mL; *p* = 0.583), or T1D and T2D (3.66 ng/mL versus 2.62 ng/mL; *p* = 0.151). In addition, there were no significant differences in sCD36 levels divided in quartiles among the three groups (*p* = 0.062) (Table 1). 

Furthermore, despite median, sCD36 concentrations were similar in women and men with T2D (2.99 ng/mL versus 2.15 ng/mL; *p* = 0.374), or T1D (3.69 ng/mL versus 3.58 ng/mL; *p* = 0.812), median sCD36 concentrations were significantly higher in women in the non-diabetic group (3.40 ng/mL versus 2.36 ng/mL; *p* = 0.047). In addition, sCD36 concentrations tended to be increased in T2D statin users (1.88 ng/mL versus 3.53 ng/mL; *p* = 0.084) and tended to be decreased in T1D statin users (4.53 ng/mL versus 2.70 ng/mL; *p* = 0.145).

### 3.2. Associations of Circulating sCD36 in the Study Groups

**Figure 1:**
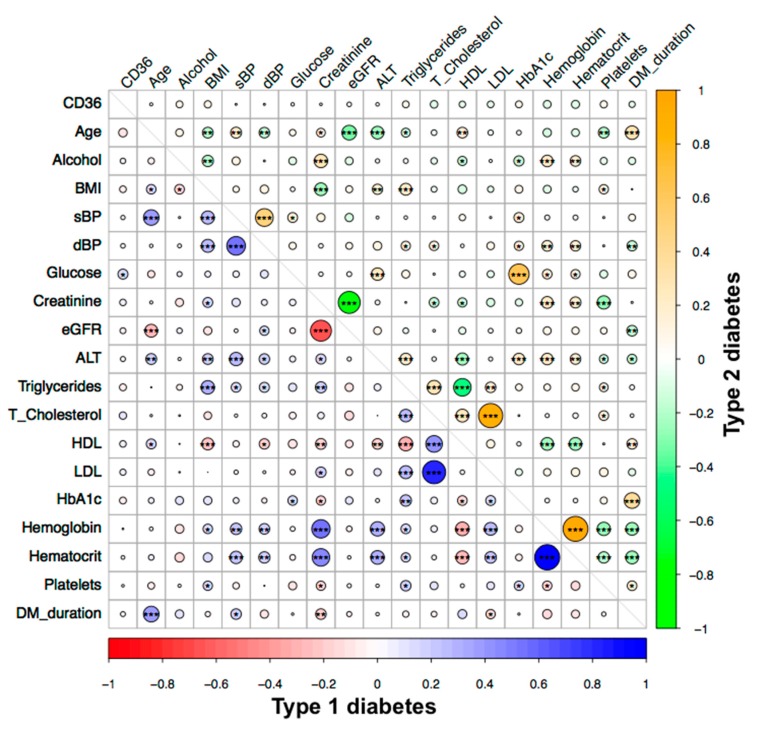
Correlation analysis of circulating sCD36 and clinical variables in type 1 diabetes (T1D) (red–blue) and type 2 diabetes (T2D) (green–orange) groups. Spearman’s correlation is significant at the 0.05 *, 0.01 **, and 0.001 *** levels (2-tailed). BMI, Body mass index; sBP, systolic blood pressure; dBP, diastolic blood pressure; eGFR, estimated glomerular filtration rate (MDRD4_IDMS equation); ALT, alanine aminotransferase; HDL, high-density lipoprotein; LDL, low-density lipoprotein; HbA1c, glycated haemoglobin; DM duration, diabetes duration.

When considering all three groups together, age, body mass index (BMI), systolic blood pressure, triglycerides, and haematocrit were negatively correlated with sCD36 (*p*-values between *p* < 0.05 and *p* < 0.01). Moreover, in the control group, age, BMI, systolic blood pressure, triglycerides, glucose, and HbA1c were negatively correlated. The number of platelets was positively correlated with sCD36 (*p*-values between *p* < 0.05 and *p* < 0.01) (Figure S1). Finally, glucose was positively correlated with sCD36 in T1D (*p* < 0.05). In the T2D group, sCD36 was not correlated with any of the analysed variables (Figure 1). 

On the other hand, in patients with T2D, significantly higher sCD36 concentrations were found in antiplatelet users (*p* = 0.039) and in patients with dyslipidaemia (*p* = 0.048). Significantly lower sCD36 concentrations were found in T1D diabetes patients with tobacco exposure (*p* = 0.005). In non-diabetic subjects, significantly lower sCD36 concentrations were found in subjects with hypertension (*p* < 0.001), dyslipidaemia (*p* = 0.044), and statin users (*p* = 0.035) (Table 2).

### 3.3. Multinomial Logistic Regression Models of sCD36

We generated multinomial logistic regression models for sCD36 quartiles of the whole study group. Additionally, to avoid model meddling because of the group characteristics, we analysed T1D, T2D, and non-diabetic control groups separately. First quartile (Q1) was taken as a referent group and was compared with the other groups, defined as second (Q2), third (Q3), and quarter (Q4) quartile, respectively. In the whole group, the variables independently associated with sCD36 concentrations were: older age with all quartiles of sCD36 (Q2: OR = 0.978, *p* = 0.014; Q3: OR = 0.980, *p* = 0.024, and Q4: OR = 0.967, *p* < 0.001), tobacco exposure with Q4 (OR = 0.581, *p* = 0.006); T2D with all quartiles of sCD36 (Q2: OR = 2.072, *p* = 0.006; Q3: OR = 1.852, *p* = 0.020, and Q4: OR = 1.925, *p* = 0.020); and Q3 of platelets with the Q2 (OR = 1.858, *p* = 0.020) and Q4 (OR = 2.204, *p* = 0.004) (Table S1). 

In the multinomial model for T1D, the variables independently associated with sCD36 concentrations were: tobacco exposure with Q3 (OR 0.289, *p* = 0.009), and Q4 (OR = 0.352, *p* = 0.015), higher BMI with Q3 (OR = 1.125, *p* = 0.044), and higher glomerular filtration rate (eGFR) with Q2 (OR = 0.968, *p* = 0.013) (Table S2). 

In the multinomial model of T2D, MPV (mean platelet volume) was associated with Q2 (OR = 0.581, *p* = 0.027) and Q3 (OR = 0.561, *p* = 0.023), insulin treatment was associated with Q2 (OR = 5.838, *p* = 0.033), treatment with oral anti-diabetic agents was associated with Q3 (OR = 4.108, *p* = 0.029), and antiplatelet treatment was associated with Q3 (OR2.694, *p* = 0.026) (Table S3). 

Finally, in the non-diabetic group, the variables independently associated with sCD36 concentrations were: higher levels of HbA1c with all quartiles (Q2: OR = 0.243, *p* < 0.001; Q3: OR = 0.260, *p* < 0.001; and Q4: OR = 0.299, *p* = 0.002), older age and higher triglycerides with Q4 (OR = 0.975, *p* = 0.039 and OR = 0.994, *p* = 0.019 respectively), and Q3 of platelets with Q2 of sCD36 (OR = 2.414, *p* = 0.029), Q2 of platelets with Q3 of sCD36 (OR = 0.443, *p* = 0.031) and Q3 and Q4 of platelets with Q4 of sCD36 (OR = 4.318, *p* < 0.001 and OR = 2.635, *p* = 0.026, respectively) (Table S4).

### 3.4. Association of sCD36 with Type 1 and Type 2 Diabetes

**Figure 2:**
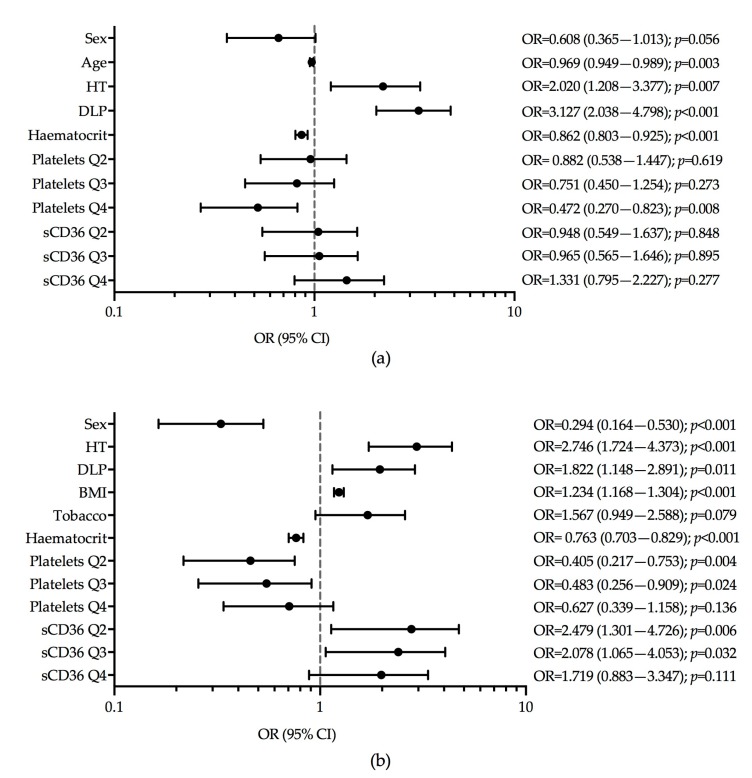
Logistic regression models for presence of diabetes. (**a**) T1D versus non-diabetic group (Hosmer and Lemeshow test *p*-value = 0.789); (**b**) T2D versus non-diabetic (Hosmer and Lemeshow test *p*-value = 0.686). Tobacco, tobacco exposure.

The multiple logistic regression model of the non-diabetic control group versus T1D showed that the variables independently associated with the presence of T1D were: older age (OR = 0.969, *p* = 0.003), the presence of hypertension (OR = 2.020, *p* = 0.007), presence of dyslipidaemia (OR = 3.127, *p* < 0.001), higher haematocrit (OR = 0.862, *p* < 0.001), and the Q4 of platelets (OR = 0.472, *p* = 0.008) (Figure 2a). The ROC curve showed non-discriminative power when sCD36 was added to the model (Figure S2a).

In the logistic regression model of the non-diabetic controls versus T2D, we observed that the variables independently associated with the presence of T2D were: female gender (OR = 0.294, *p* < 0.001), hypertension (OR = 2.746, *p* < 0.001), dyslipidaemia (OR = 1.822, *p* = 0.011), high BMI (OR = 1.234, *p* < 0.001), higher haematocrit (OR = 0.763, *p* < 0.001), Q2 and Q3 of platelets (OR = 0.405, *p* = 0.004 and OR = 0.483, *p* = 0.024 respectively), and Q2 and Q3 of sCD36 (OR = 2.479, *p* = 0.006 and OR = 2.078, *p* = 0.032, respectively) (Figure 2b). The models with and without sCD36 showed that the addition of sCD36 concentrations did not provide additional discriminative power to the model area under the ROC curve of 0.844, *p* < 0.001 versus 0.851, *p* < 0.001 (Figure S2b).

### 3.5. Flow Cytometric Analysis of CD36 and Real-Time PCR

Flow cytometric analysis was used to detect the expression of CD36 in circulating mononuclear cells from T2D patients and non-diabetic control subjects. In the recruited group, the mean age was 55 years, and up to 46% were men. No significant differences were found in the expression of CD36 scavenger receptor either in monocytes, or in leukocyte population between T2D patients and the non-diabetic group (Figure S3). Regarding mRNA expression, no differences in CD36 expression were found between T2D patients and the non-diabetic control group (Figure S4).

### 3.6. Consistency Test in sCD36 Assessment

In the assay of sCD36, performed in healthy volunteers, we did not find differences in sCD36 among the different centrifugation forces or among the number of freeze-thaw cycles. Paired plasma samples prepared by 1500× *g*, 1850× *g*, and 3000× *g* gave nearly identical results and a good correlation within each assay. Moreover, in the assays that compared samples with three, or without, freeze-thaw cycles, there were no differences in the results of the three different centrifugation forces, which were highly correlated (Table S5). 

## 4. Discussion

In the present study, we did not observe differences in plasma sCD36 concentrations between patients with T1D, T2D, and non-diabetic control subjects, all of them with normal renal function and free from advanced late diabetic complications, including macrovascular disease. Overall, there was an association between sCD36 plasma concentrations and cardiovascular risk factors such as hypertension, dyslipidaemia, tobacco exposure, age, sex, and BMI. In the current study, we measured most of the variables that have been previously described to be associated with sCD36. These factors included the use of several medications, age, sex, systolic and diastolic blood pressure, tobacco exposure, BMI, HDL, LDL, total cholesterol, triglycerides, haematocrit, haemoglobin, platelets, serum creatinine, blood glucose, and HbA1c. In addition, we have assessed for the first time, the contribution of all of these factors together in a large group of subjects with and without diabetes. Importantly, we found increased sCD36 in patients with T2D that had dyslipidaemia or that were antiplatelet users. As dyslipidaemia and the use of antiplatelet drugs is relatively frequent in T2D, this could have led to spuriously elevated sCD36 in previous studies in patients with T2D.

Regarding the correlations between sCD36 concentrations and clinical variables, such as components of the metabolic syndrome among them insulin resistance, there is a large variability [20,21,23,24,32,33]. In the non-diabetic group, sCD36 concentrations were negatively correlated with glucose and HbA1c, and positively correlated with the number of platelets, as already described in the literature. However, we found a negative correlation of sCD36 with age, BMI, and triglycerides, in contrast to the reported findings of a previous study [21,32,34]. Interestingly, and in accordance with the findings of another study, we found no significant correlations between sCD36 with all variables analysed in T2D [24]. This may explain, at least in part, that our results are discordant with some of the previous studies that found an association of sCD36 with insulin resistance, components of the metabolic syndrome, and the risk of T2D [20,32,35]. An additional reason for these differences may lie in the diverse characteristics of the study populations, which include differences in age, duration of diabetes, metabolic syndrome components, the presence of complications, and the use of antidiabetic medications. The characteristics of our population study faithfully reflects the characteristics of the Spanish population [36]. Regarding medication, and in contrast to the findings reported, we did not find differences in sCD36 concentrations between diabetic patients with or without statin medication [26]. Furthermore, the number of subjects with diabetes in our study was larger than that in previous studies and included patients with T1D for the first time. Previous studies with a lower number of subjects found higher levels of sCD36 in subjects with T2D [20,21,35]. However, in a subsequent study with 200 T2D patients, although these subjects exhibited higher sCD36 concentrations, the only characteristics that predicted sCD36 were diabetes duration and markers of platelet activation [34]. The latter findings are in line with ours. 

To our knowledge, this is the first study that addresses the potential association of sCD36 with diabetes as a primary study objective. We did not find a clear association of sCD36 concentrations with T2D, the evaluation of the ROC curve shows that sCD36 does not contribute additional power to the predictive model. In fact, we did find a weak association of sCD36 with T2D, which may partly be due to the clinical and metabolic characteristics, among them waist circumference, which is an indirect measure of insulin resistance. In order to demonstrate that insulin resistance per se may be an associated factor to sCD36, direct measurements of insulin resistance should have been used. Regarding T1D, we found no association with sCD36. Patients with T1D showed younger age and had a high-frequency of hypertension and dyslipidaemia. Since sCD36 concentrations were not related to T1D, this may indicate indirectly that hyperglycaemia, which is a common feature in patients with either T1D or T2D, is not one of the main factors contributing to plasma sCD36 concentrations. Our findings are in line with some recent studies that found no association of sCD36 with diabetes or its associated metabolic disturbances in middle-aged subjects with metabolic syndrome [24], or patients with early-onset coronary artery disease [25]. Another study found that baseline sCD36 did not predict diabetes independently of fasting glucose and insulin in a non-diabetic population [22]. In that study, the addition of sCD36 in a multivariate model of diabetes prediction had no impact on diabetes prediction. 

CD36 signalling responds to ox-LDL, and on the one hand, reduces macrophage motility and probably induces the trapping of macrophages in the arterial intima, which promotes atherosclerosis. On the other hand, it induces chronic inflammation and contributes to insulin resistance common in obesity and dyslipidaemia, both associated to metabolic disorders [37,38]. However, this does not seem to be consistently associated with its circulating levels. Regarding the determination of circulating sCD36, there is a lack of well-characterized or standardized methods to assess its concentrations [30]. Despite this scarcity, as reported previously [30], the pre-analytical procedures do not explain the variability of the results reported in different studies. Differences between studies may be explained by several factors: (a) regarding the heterogeneous origin of CD36 particles in the plasma of patients [39,40], our data show no differences in the CD36 surface expression from monocytes and leukocytes of peripheral blood among patients with T2D under antidiabetic treatment and non-diabetic subjects. (b) Although hyperglycaemia was shown to influence CD36 mRNA expression increasing the de novo CD36 synthesis in monocytes from healthy subjects [41], we did not find differences in the CD36 mRNA expression of monocytes and leukocytes from peripheral blood between non-diabetic subjects and patients with T2D under anti-diabetic treatment. (c) In poorly-controlled patients, lower plasma levels of glycated CD36 and higher levels of non-glycated CD36 have been described in comparison with well-controlled diabetic patients [39], and this fact may account also for differences with different assays. (d) Another condition that could influence the concentration of CD36 is the number of microparticles to which it is associated, e.g., CD36 associated with platelet-derived microparticles may be increased in proportion to these cell particles in non-diabetic subjects [40,42]. Actually, our findings showed that increased circulating platelet numbers are independently associated with a moderate and a high-sCD36 concentration in non-diabetic subjects. 

Our study has some limitations. First, although we adjusted for risk factors known to be associated with diabetes in our analyses, the possibility that some other confounding factors play a role may have been incompletely accounted for in plasma sCD36. Second, we did not validate our assay with tests from other manufacturers. Third, the cross-sectional design precludes conclusions about causality, and therefore prospective studies are needed to establish the usefulness of the sCD36 plasma concentration as a predictive factor for T1D and T2D. Finally, as we did not measure insulin resistance in the study subjects, this precludes the assessment of the impact of this metabolic variable on plasma sCD36 concentration.

## 5. Conclusions

Circulating plasma sCD36 concentrations do not appear to be a biomarker of T1D or T2D. However, our study does not completely rule out the possibility that sCD36 may constitute a biomarker of cardiovascular events in diabetes and other populations. The follow-up of the cohorts with and without diabetes subjects may help to clarify this question. 

## Figures and Tables

**Table 1:** Clinical and anthropometrical characteristics of the study groups.

Variables ^1^	Control	T1D	T2D	*p* Value	*p* Control versus T1D	*p* Control versus T2D	*p* T1D versus T2D
	*N* = 522	*N* = 225	*N* = 276				
Sex, Men	212 (40.6%)	109 (48.4%)	143 (51.8%)	0.006	0.086	0.009	0.509
Age, years	49 (39–59)	44 (38.0–51.0)	59 (51–66)	<0.001	<0.001	<0.001	<0.001
BMI, Kg/m^2^	25.5 (23.4–28.3)	25.6 (22.6–28.1)	30.2 (27.9–34.7)	<0.001	0.557	<0.001	<0.001
Waist, cm	94.0 (85.0–102)	88.0 (80.0–97.0)	104 (97.5–112)	<0.001	<0.001	<0.001	<0.001
Alcohol mg/day	2.86 (0–12.3)	2.37 (0–7.19)	1.53 (0–9.92)	0.030	0.095	0.055	0.685
Tobacco	270 (51.7%)	116 (51.6%)	149 (54.0%)	0.805	1.000	0.977	0.977
Hypertension	81 (16%)	55 (24.4%)	153 (55.4%)	<0.001	0.009	<0.001	<0.001
sBP, mmHg	121 (110–132)	128 (116–138)	139 (127–150)	<0.001	<0.001	<0.001	<0.001
dBP, mmHg	76.0 (70–83)	75.0 (68.5–80)	76.0 (70–84)	0.020	0.026	0.889	0.028
Dyslipidaemia	115 (22.6%)	96 (42.7%)	129 (46.7%)	<0.001	<0.001	<0.001	0.412
Antiplatelet	-	63 (28%)	99 (35.9%)	<0.001	-	-	0.076
Statins	114 (22.4%)	96 (42.7%)	116 (42%)	<0.001	<0.001	<0.001	0.958
Glucose, mg/dL	90 (84–96)	159 (107–208)	148 (117–182)	<0.001	<0.001	<0.001	0.619
HbA1c, %	5.5 (5.2–5.8)	7.40 (7.00–7.9)	7.60 (6.80–8.5)	<0.001	<0.001	<0.001	0.230
Creatinine mg/dL	0.79 (0.70–0.93)	0.78 (0.65–0.87)	0.79 (0.68–0.93)	0.011	0.009	0.567	0.046
eGFR	86.2 (77.4–97.6)	95.2 (82.7–108)	86.8 (77.5–100)	<0.001	<0.001	0.383	<0.001
ALT U/L	17 (13–23)	17 (13.8–21)	19 (16–28)	<0.001	0.719	<0.001	<0.001
Triglycerides, mg/dL	87 (67.0–125)	65 (53–86)	118 (84.8–166)	<0.001	<0.001	<0.001	<0.001
Total-C, mg/dL	196 (175–226)	180 (164–201)	184 (163–206)	<0.001	<0.001	<0.001	0.180
HDL, mg/dL	57 (48–68)	62.5 (53–74)	48 (40–58)	<0.001	<0.001	<0.001	<0.001
LDL, mg/dL	119 (100–142)	101 (84.5–116)	108 (88.4–129)	<0.001	<0.001	<0.001	0.003
Haemoglobin, g/dL	14.2 (13.3–15.2)	14 (13.1–14.9)	13.7 (12.9–14.7)	<0.001	0.031	<0.001	0.098
Haematocrit, %	42.4 (3.58)	41.5 (3.51)	41.1 (3.54)	<0.001	0.003	<0.001	0.545
Platelets, 10^9^/L	233 (203–273)	223 (197–256)	226 (188–274)	0.019	0.027	0.078	0.759
Diabetes duration, years	-	21 (15–29)	8 (4–13.5)	<0.001	-	-	<0.001
sCD36, ng/mL	2.84 (0.56–9.06)	3.66 (0.69–18.7)	2.62 (0.66–7.57)	0.144	0.180	0.583	0.151
sCD36 Quartiles ^2^				0.062	0.259	0.259	0.036
(<−0.562)	134 (25.7%)	53 (23.6%)	64 (23.2%)				
(−0.562,1.05)	127 (24.3%)	49 (21.8%)	80 (29%)				
(1.05,2.24)	133 (25.5%)	51 (22.7%)	77 (27.9%)				
(>2.24)	128 (24.5%)	72 (32.0%)	55 (19.9%)				

^1^ Mean (SD) or median (Interquartile range) for continuous variables and frequency (percentage) for categorical variables; ^2^ Soluble CD36 in quartiles was Ln transformed. BMI, body mass index; sBP, systolic blood pressure; dBP, diastolic blood pressure; ALT, alanine aminotransferase; eGFR; estimated glomerular filtration rate (MDRD4_IDMS equation); HDL, high-density lipoprotein; LDL, low-density lipoprotein.

**Table 2:** Association of circulating sCD36 concentration and qualitative parameters in the whole study group, type 1 diabetes, type 2 diabetes, and non-diabetic subjects.

Variables		Whole Group		Control		T1D		T2D	
		Median (IQR)	*p*-Value	Median (IQR)	*p*-Value	Median (IQR)	*p*-Value	Median (IQR)	*p*-Value
Sex	Men	2.59 (0.48–8.51)	0.089	2.36 (0.36–8.06)	0.047	3.58 (0.69–27.9)	0.812	2.15 (0.65–6.29)	0.374
	Women	3.37 (0.75–9.99)		3.40 (0.74–10.3)		3.69 (0.71–15.1)		2.99 (0.82–8.28)	
Hypertension	No	3.17 (0.69–10.9)	0.024	3.23 (0.73–9.93)	0.001	3.57 (0.70–20.7)	0.751	2.29 (0.64–8.39)	0.875
	Yes	2.65 (0.48–7.17)		1.04 (0.17–5.10)		3.72 (0.68–8.79)		2.83 (0.79–6.14)	
Dyslipidaemia	No	3.09 (0.66–10.3)	0.274	3.15 (0.69–10.0)	0.044	4.53 (0.85–28.5)	0.090	1.80 (0.48–6.95)	0.048
	Yes	2.79 (0.51–8.33)		2.04 (0.26–7.80)		2.75 (0.44–9.89)		3.53 (1.01–7.95)	
Statins	No	3.09 (0.66–10.1)	0.247	3.15 (0.69–9.93)	0.035	4.53 (0.84–23.7)	0.145	1.88 (0.48–6.88)	0.084
	Yes	2.74 (0.48–8.32)		2.01 (0.26–7.64)		2.70 (0.42–13.1)		3.54 (1.02–8.03)	
Obesity	No	3.15 (0.65–10.2)	0.130	3.12 (0.69–9.59)	0.030	3.58 (0.65–24.4)	0.644	2.51 (0.65–7.08)	0.546
	Yes	2.43 (0.53–7.59)		1.70 (0.26–5.91)		3.81 (0.84–10.4)		2.73 (0.81–7.86)	
Tobacco	No	3.68 (0.82–10.5)	0.003	2.92 (0.64–9.58)	0.321	6.38 (1.16–25.2)	0.005	3.24 (0.94–8.58)	0.176
	Yes	2.55 (0.48–7.94)		2.60 (0.48–8.17)		2.37 (0.05–10.9)		2.14 (0.65–6.38)	
Anti-platelet	No	2.86 (0.56–9.55)	0.393	-	-	4.29 (0.68–19.2)	0.410	1.98 (0.48–6.81)	0.039
	Yes	3.27 (0.88–8.42)		-		2.66 (0.73–17.8)		3.59 (1.07–8.06)	
Diabetes	No	2.84 (0.56–9.06)	0.597	-	-	-	-	-	-
	Yes	2.99 (0.67–9.52)		-		-		-	

## Supplementary Materials

The following are available online at https://www.mdpi.com/2077-0383/8/5/710/s1, Table S1: Multinomial regression model for circulating sCD36 in the whole study group. Table S2. Multinomial regression model for sCD36 in type 1 diabetic group. Table S3. Multinomial regression model for sCD36 in type 2 diabetic group. Table S4. Multinomial regression model for sCD36 in the non-diabetic control group. Table S5. Comparison of pre-analytical conditions of sCD36. Figure S1. Correlation analysis of circulating sCD36 and clinical variables in whole (red–blue) and non-diabetic subject (green–orange) groups. Figure S2. Receiver operating characteristics (ROC) curve showing the relationship between sensitivity and 1-specificity in determining the discriminatory ability of the logistic regression model with and without sCD36 as predictor. Figure S3. Ex vivo flow cytometric analysis of CD36 from type 2 diabetic patients and non-diabetic subjects. Figure S4. CD36 mRNA expression was not different between T2D and non-diabetic controls.
