# Supplementary material for: Circulating Soluble CD36 is Similar in Type 1 and Type 2 Diabetes Mellitus versus Non-Diabetic Subjects
Source: J Clin Med. 2019 May 18;8(5):710. doi: 10.3390/jcm8050710 (PMC6571689; doi:10.3390/jcm8050710)
Supplement: Supplementary file 1 [file jcm-08-00710-s001.pdf]

Table S1. Multinomial regression model for circulating sCD36 in the whole study group.

	**sCD36 Quartile 2**			**sCD36 Quartile 3**			**sCD36 Quartile 4**		
	**OR**	**95% CI**	***p***	**OR**	**95% CI**	***p***	**OR**	**95% CI**	***p***
Age	0.978	0.960–0.995	**0.014**	0.980	0.963–0.997	**0.024**	0.967	0.950–0.985	**<0.001**
Sex, female	1.172	0.775–1.772	0.451	1.342	0.889–2.027	0.162	1.029	0.676–1.566	0.894
Hypertension	0.809	0.514–1.272	0.358	1.012	0.647–1.582	0.958	0.657	0.408–1.060	0.085
Tobacco exposure	0.736	0.498–1.087	0.124	0.709	0.481–1.045	0.082	0.581	0.394–0.857	**0.006**
BMI	0.987	0.948–1.028	0.536	0.963	0.924–1.004	0.074	0.970	0.930–1.012	0.157
ALT	1.008	0.998–1.019	0.116	1.002	0.990–1.014	0.715	0.987	0.971–1.003	0.110
Type 1 diabetes	1.019	0.608–1.707	0.941	0.813	0.488–1.354	0.427	1.174	0.720–1.913	0.520
Type 2 diabetes	2.072	1.234–3.477	**0.006**	1.852	1.101–3.114	**0.020**	1.925	1.109–3.341	**0.020**
Systolic blood pressure	1.002	0.990–1.014	0.690	1.005	0.993–1.017	0.411	1.002	0.989–1.014	0.787
Triglycerides	0.999	0.997–1.001	0.325	0.997	0.994–1.00	0.057	0.999	0.997–1.001	0.433
LDL cholesterol	1.005	0.998–1.011	0.144	0.999	0.993–1.006	0.917	0.997	0.991–1.004	0.450
Platelets Quartile 2	0.915	0.549–1.522	0.731	0.662	0.400–1.092	0.106	1.481	0.889–2.469	0.132
Platelets Quartile 3	1.858	1.103–3.127	**0.020**	1.180	0.704–1.979	0.530	2.204	1.278–3.802	**0.004**
Platelets Quartile 4	1.393	0.811–2.391	0.230	1.254	0.746–2.108	0.392	1.738	0.991–3.049	0.054

Significant values are shown in bold. In the multinomial regression model, sCD36 was Ln transformed, and the first quartile was used as the reference. BMI, body mass index; ALT, alanine aminotransferase; LDL, low-density lipoprotein.

Table S2. Multinomial regression model for sCD36 in type 1 diabetic group.

	**sCD36 Quartile 2**			**sCD36 Quartile 3**			**sCD36 Quartile 4**		
	**OR**	**95% CI**	***p***	**OR**	**95% CI**	***p***	**OR**	**95% CI**	***p***
Age	0.992	0.943–1.043	0.757	1.035	0.985–1.087	0.168	0.971	0.927–1.074	0.218
Sex, female	1.720	0.669–4.426	0.260	0.782	0.299–2.045	0.617	0.878	0.369–2.086	0.768
Body mass index	1.111	0.989–1.248	0.076	1.125	1.003–1.262	**0.044**	1.031	0.927–1.148	0.571
Triglycerides	1.015	0.941–1.095	0.701	0.900	0.666–1.214	0.489	0.899	0.666–1.213	0.488
Platelets Quartile 2	1.635	0.484–5.526	0.429	0.759	0.239–2.408	0.640	2.024	0.725–5.651	0.178
Platelets Quartile 3	1.570	0.472–5.223	0.462	0.759	0.231–2.486	0.647	0.889	0.290–2.727	0.837
Platelets Quartile 4	2.123	0.555–8.123	0.271	1.224	0.341–4.397	0.756	1.310	0.376–4.567	0.672
Tobacco exposure	0.493	0.192–1.265	0.141	0.289	0.144–0.731	**0.009**	0.352	0.152–0.817	**0.015**
Antiplatelet treatment	2.958	1.027–8.522	**0.044**	0.614	0.199–1.895	0.396	1.508	0.550–4.136	0.425
eGFR	0.968	0.944–0.993	**0.013**	0.989	0.968–1.012	0.359	0.988	0.968–1.007	0.228
Total cholesterol	0.876	0.596–1.286	0.498	1.676	0.374–7.508	0.500	1.059	0.353–7.124	0.544
HDL cholesterol	1.124	0.765–1.652	0.551	0.606	0.135–2.715	0.513	0.632	0.141–2.831	0.549
LDL cholesterol	1.172	0.798–1.723	0.418	0.608	0.136–2.725	0.516	0.644	0.144–2.876	0.563

Significant values are shown in bold. In the multinomial regression model, sCD36 was Ln transformed, and the first quartile was used as the reference. eGFR; estimated glomerular filtration rate (MDRD4_IDMS equation).

Table S3. Multinomial regression model for sCD36 in type 2 diabetic group.

	sCD36 Quartile 2			sCD36 Quartile 3			sCD36 Quartile 4		
	**OR**	**95% CI**	***p***	**OR**	**95% CI**	***p***	**OR**	**95% CI**	***p***
Age	0.966	0.928–1.007	0.101	0.999	0.958–1.042	0.980	0.991	0.947–1.037	0.687
Sex, female	0.753	0.322–1.762	0.513	1.497	0.620–3.615	0.369	1.022	0.395–2.637	0.965
Platelets Quartile 2	1.321	0.436–4.002	0.622	0.971	0.299–3.151	0.961	1.855	0.566–6.081	0.307
Platelets Quartile 3	0.996	0.365–2.715	0.994	0.729	0.256–2.081	0.555	1.119	0.357–3.508	0.846
Platelets Quartile 4	0.953	0.310–2.923	0.933	1.083	0.363–2.233	0.886	1.045	0.296–3.695	0.945
Antiplatelet treatment	2.099	0.880–5.009	0.945	2.694	1.126–6.447	**0.026**	1.867	0.706–4.439	0.208
Total cholesterol	1.010	0.984–1.036	0.471	0.971	0.936–1.007	0.113	1.001	0.972–1.031	0.936
HDL cholesterol	1.006	0.974–1.038	0.715	1.022	0.984–1.060	0.255	0.983	0.948–1.020	0.382
LDL cholesterol	0.997	0.968–1.026	0.824	1.031	0.990–1.073	0.135	0.990	0.958–1.024	0.577
Mean platelet volume	0.581	0.359–0.940	**0.027**	0.561	0.341–0.924	**0.023**	0.668	0.388–1.148	0.144
Treatment OAD	2.899	0.854–9.846	0.088	4.108	1.157–11.458	**0.029**	1.956	0.483–7.920	0.347
Treatment OAD + Insulin	2.448	0.673–8.907	0.174	2.589	0.670–9.998	0.167	2.607	0.600–11.331	0.201
Treatment Insulin	5.838	1.152–29.583	**0.033**	5.068	0.930–27.606	0.060	3.634	0.558–23.663	0.176
Treatment Other	2.006	0.478–8.424	0.341	2.345	0.513–10.719	0.271	4.543	0.947–21.795	0.058

Significant values are shown in bold. In the multinomial regression model, sCD36 was Ln transformed, and the first quartile was used as the reference. OAD, Oral Antidiabetic Agents; HDL, high-density lipoprotein; LDL, low-density lipoprotein.

Table S4. Multinomial regression model for sCD36 in the non-diabetic control group.

	**sCD36 Quartile 2**			**sCD36 Quartile 3**			**sCD36 Quartile 4**		
	**OR**	**95% CI**	***p***	**OR**	**95% CI**	***p***	**OR**	**95% CI**	***p***
Age	0.991	0.967–1.014	0.436	0.985	0.962–1.008	0.201	0.975	0.952–0.999	**0.039**
Sex, female	1.081	0.528–2.211	0.831	1.291	0.632–2.636	0.483	1.310	0.636–2.694	0.463
Hypertension	0.568	0.269–1.200	0.138	0.574	0.270– 1.222	0.150	0.574	0.264–1.245	0.160
Dyslipidaemia	0.862	0.459–1.620	0.645	0.917	0.493–1.704	0.784	0.830	0.435–1.585	0.573
Body mass index	0.998	0.935–1.067	0.966	0.957	0.893–1.025	0.212	0.984	0.921–1.052	0.642
Triglycerides	0.996	0.992–1.000	0.081	0.997	0.993–1.001	0.150	0.994	0.989–0.999	**0.019**
HbA1c	0.243	0.112–0.523	**<0.001**	0.260	0.121–0.557	**<0.001**	0.299	0.138–0.648	**0.002**
Platelets Quartile 2	0.653	0.302–1.409	0.277	0.443	0.212–0.927	**0.031**	1.136	0.501–2.573	0.760
Platelets Quartile 3	2.414	1.094–5.322	**0.029**	1.475	0.685–3.174	0.320	4.318	1.855–1.005	**<0.001**
Platelets Quartile 4	1.497	0.669–3.350	0.326	1.029	0.476–2.224	0.942	2.635	1.120–6.197	**0.026**
Haematocrit	0.948	0.856–1.049	0.299	0.972	0.879–1.075	0.586	1.006	0.909–1.114	0.904

Significant values are shown in bold. In the multinomial regression model, sCD36 was Ln transformed, and the first quartile was used as the reference. HbA1c, glycated haemoglobin.

Table S5. Comparison of pre-analytical conditions of sCD36.

	**Centrifugation 3000× *g***		**Centrifugation 1850× *g***		**Centrifugation 1500× *g***	
	**1 Freeze-Thaw Cycle**	**3 Freeze-Thaw Cycle**	**1 Freeze-Thaw Cycle**	**3 Freeze-Thaw Cycle**	**1 Freeze-Thaw Cycle**	**3 Freeze-Thaw Cycle**
Centrifugation 3000× *g*						
1 freeze-thaw cycle	1	0.974 [1]	0.997 [1]	0.945 [1]	0.998 [1]	0.983 [1]
3 freeze-thaw cycle		1	0.969 [1]	0.914 [1]	0.973 [1]	0.974 [1]
Centrifugation 1850× *g*						
1 freeze-thaw cycle			1	0.927 [1]	0.999 [1]	0.980 [1]
3 freeze-thaw cycle				1	0.933 [1]	0.954 [1]
Centrifugation 1500× *g*						
1 freeze-thaw cycle					1	0.983 [1]
3 freeze-thaw cycle						1

[1] Pearson's correlation is significant at the 0.01 level (2-tailed). Pearson correlation coefficients between pre-analytical steps, measured within each assay. Correlation between plasma prepared at 3000× *g*, 1850× *g* and 1500× *g* centrifugation, everyone with the correlation between plasma treated by 1 and 3 freeze-thaw cycles.

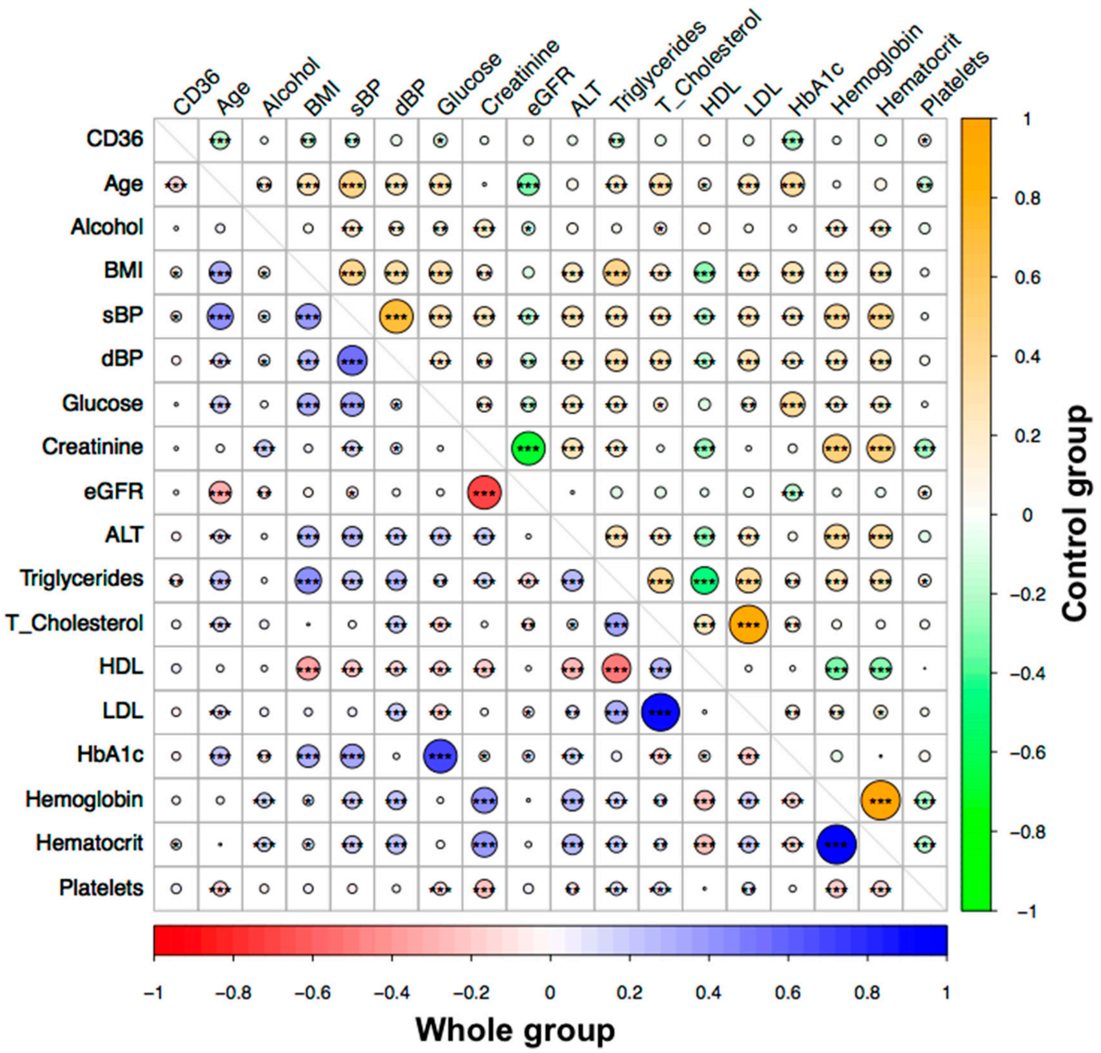

Figure S1. Correlation analysis of circulating sCD36 and clinical variables in whole (red–blue) and non-diabetic subject (green–orange) groups. Spearman's correlation is significant at the 0.05 *, 0.01 ** and 0.001*** levels (2-tailed). BMI, body mass index; sBP, systolic blood pressure; dBP, diastolic blood pressure; eGFR, estimated glomerular filtration rate (MDRD4_IDMS equation); ALT, alanine aminotransferase; HDL, high-density lipoprotein; LDL, low-density lipoprotein.

Diabetes model in T1D

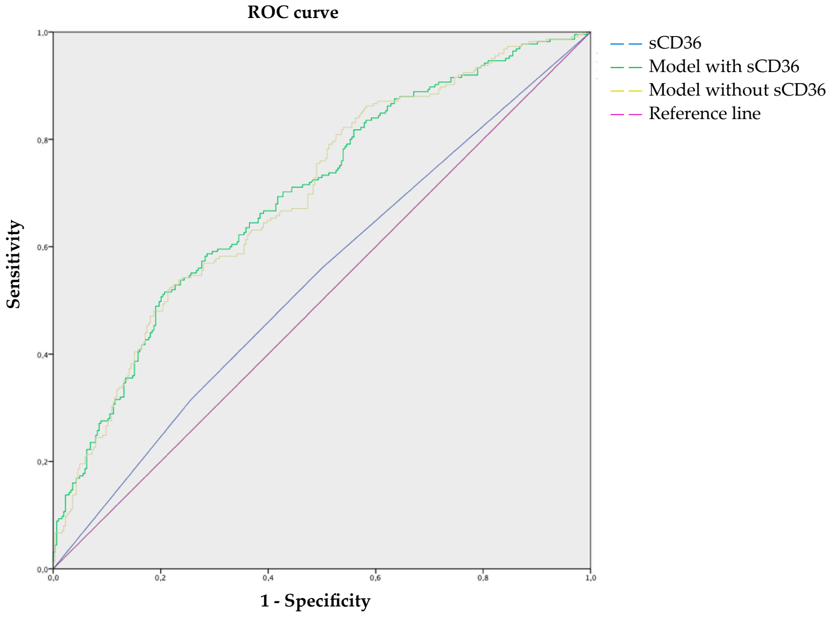

Test Results Variables	Area	Std. Error	Asymptotic Sig.	Asymptotic 95% Confidence Interval	
				Lower Bound	Upper Bound
sCD36	0.537	0.025	143	0.488	0.587
Model with sCD36	0.695	0.023	0	0.650	0.740
Model without sCD36	0.694	0.023	0	0.649	0.739

(a)

Diabetes model in T2D

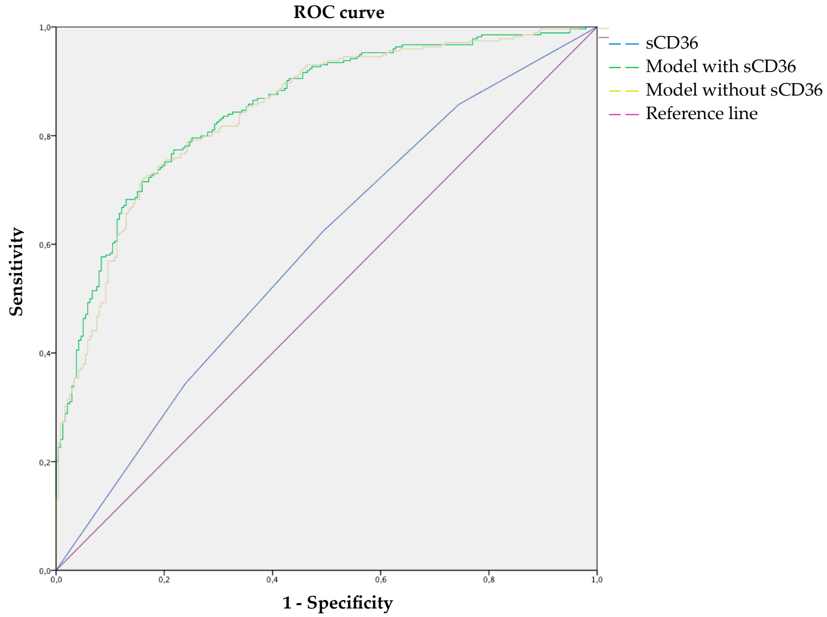

Test Results Variables	Area	Std. Error	Asymptotic Sig.	Asymptotic 95% Confidence Interval	
				Lower Bound	Upper Bound
sCD36	0.587	0.025	0.001	0.538	0.637
Model with sCD36	0.851	0.017	0	0.819	0.884
Model without sCD36	0.844	0.017	0	0.811	0.878

(b)

Figure S2. Receiver operating characteristics (ROC) curve showing the relationship between sensitivity and 1-specificity in determining the discriminatory ability of the logistic regression model with and without sCD36 as predictor. (**a**) T1D versus non-diabetic group, and (**b**) T2D versus non-diabetic group.

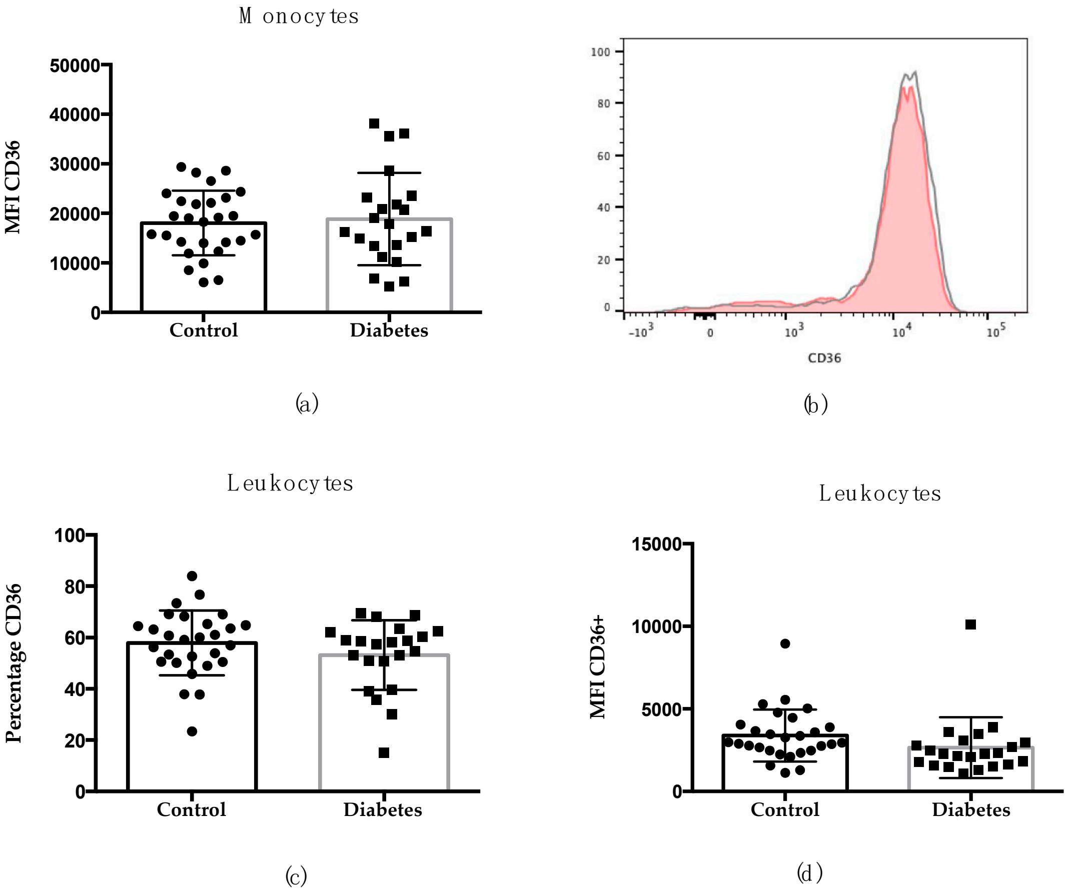

Figure S3. Ex vivo flow cytometric analysis of CD36 from type 2 diabetic patients and non-diabetic subjects. (**a**) CD36 median fluorescence intensity (MFI) in monocyte population. (**b**) Relative cells number CD36+ in non-diabetic subjects (in grey) and in T2D subjects (in red). (**c**) Abundance of CD36+ leukocyte population. (**d**) CD36 median fluorescence intensity (MFI) in leukocyte population.

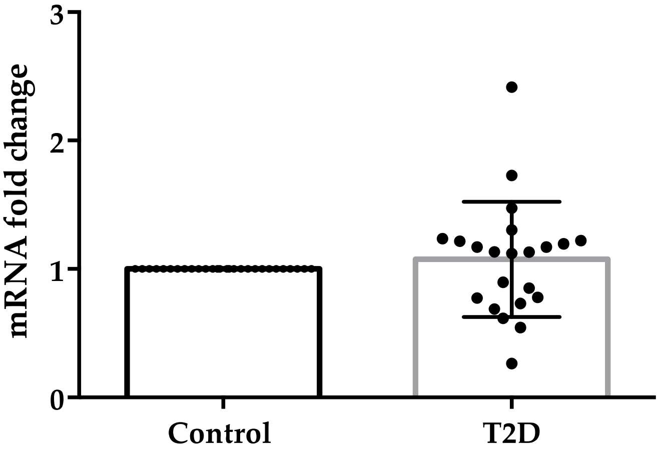

Figure S4. CD36 mRNA expression was not different between T2D and non-diabetic controls. CD36 mRNA expression was analysed by real-time PCR and normalised to GAPDH. The data show the mean of fold change relative control group from the analyses' subjects. Mann–Whitney test $p = 0.232$.
